# Sex-Specific Effects of Cardiovascular Risk Factors on Endothelium-Dependent Dilation and Endothelin Activity in Middle-Aged Women and Men

**DOI:** 10.1371/journal.pone.0121810

**Published:** 2015-03-25

**Authors:** Vijaywant Brar, Sartaj Gill, Carmine Cardillo, Manfredi Tesauro, Julio A. Panza, Umberto Campia

**Affiliations:** 1 MedStar Cardiovascular Research Network and MedStar Washington Hospital Center, Washington, District of Columbia, United States of America; 2 Prince George's Hospital Center, Cheverly, Maryland, United States of America; 3 Internal Medicine, Catholic University, Rome, Italy; 4 Systems Medicine, University of Tor Vergata, Rome, Italy; 5 Westchester Medical Center, Valhalla, New York, United States of America; University of Bologna, ITALY

## Abstract

**Background:**

Healthy middle-aged postmenopausal women have higher endothelium-dependent dilation and lower vasoconstrictor activity of endothelin-1 than men. Whether these sex-specific differences extend to patients with cardiovascular risk factors has not been investigated. The current study aimed to determine whether, in patients with cardiovascular risk factors, sex-specific differences exist in endothelium-dependent dilation and endothelin-1 activity.

**Methods:**

Forearm blood flow responses were measured by strain-gauge plethysmography during the intra-arterial infusion of acetylcholine, sodium nitroprusside, and the selective endothelin type A receptor blocker BQ-123 in 50 women and 64 men with cardiovascular risk factors.

**Results:**

Acetylcholine and sodium nitroprusside induced a significant vasodilation in women and men alike (p < 0.01 for both). Also BQ-123 caused a significant vasodilation (p < 0.001) in both groups. The vasodilator response to acetylcholine was greater in women compared to men; however there were no differences in the response to sodium nitroprusside and BQ-123 (p = NS for both) between the two sex groups.

**Conclusions:**

Middle-aged women with cardiovascular risk factors have significantly higher endothelium-dependent dilation than middle-aged men; however, vascular endothelin 1 activity is similar in the two groups. These findings suggest that the presence of cardiovascular risk factors is associated with sex-specific effects on endothelium-dependent dilation but not on endothelin 1 activity. Further study is needed to confirm our findings and to characterize the mechanisms underlying this sex-specific regulation of endothelial function.

## Introduction

Epidemiological evidence indicates that, compared with men, healthy middle-aged women have a lower estimated 10-year cardiovascular disease risk [1]. However, this sex-specific difference in risk appears to progressively diminish in the presence of one or more major cardiovascular risk factors (CRFs) [1].

We and others have demonstrated that patients with major CRFs have endothelial dysfunction, a reactive vascular phenotype considered the earliest detectable stage of atherosclerosis [2]. Endothelial dysfunction is characterized by impaired endothelium-dependent dilation (EDD) secondary to reduced bioactivity of the vasodilator molecule nitric oxide (NO) [3], and increased vascular tone mediated by the potent vasoconstrictor peptide endothelin-1 (ET-1) [4–6]. Importantly, in patients with CRFs, endothelial dysfunction is associated with an increased risk of future cardiovascular events[7].

Previous data indicate that healthy middle-aged women have higher EDD [8] and lower ET-1 activity compared to men [9], a potential mechanism underlying the lower cardiovascular risk observed in this population. However, the possible influence of CRFs on the sex-specific differences in endothelial function has not been investigated. Therefore, the current study was designed to determine whether major CRFs modify the differences that exist in EDD and vascular ET-1 activity between middle-aged women and men.

## Methods

### Study Participants

The study population included middle-aged (40 to 65 years old) men and postmenopausal women with a diagnosis of essential hypertension (systolic blood pressure ≥140 mm Hg or diastolic blood pressure ≥90 mm Hg), or hypercholesterolemia (total cholesterol ≥240 mg/dL), or type 2 diabetes mellitus (fasting blood glucose ≥ 126 mg/dL) who participated in prospective studies designed to investigate in vivo endothelial function (EDD and ET-1 activity) and conducted at the Vascular Physiology Laboratory of the MedStar Washington Hospital Center. Patients with hypertension were excluded if they also had a diagnosis of hypercholesterolemia or diabetes mellitus; patients with hypercholesterolemia were excluded if they also had a diagnosis of hypertension or diabetes. A diagnosis of hypertension or hypercholesterolemia was not an exclusion criterion in patients with type 2 diabetes. None of the patients had a history of coagulopathy or any disease predisposing them to vasculitis or Raynaud’s phenomenon. Each subject underwent screening including a detailed medical history and physical examination, ECG, complete blood count, and chemistry panel. Insulin sensitivity was assessed using the validated QUICKI method [10]. Participants on statins were asked to stop taking them at least 1 month before the study. Antihypertensive medications were withdrawn under monitoring at least 2 weeks before the study. Patients were excluded if they had clinical evidence of any disease that could affect participation in the study or its results. The study protocols were approved by the MedStar Research Institute Investigational Review Board and all patients gave written informed consent.

### Protocols

Studies were performed in the morning in a quiet room with a temperature of approximately 22°C. Participants were asked to refrain from smoking, drinking alcohol or beverages containing caffeine for at least 24 hours before the studies. Each study consisted of infusion of drugs into the brachial artery and measurement of forearm blood flow (FBF) by means of strain gauge plethysmography, as previously described in detail [11]. All drugs given in this study were approved for human use by the Food and Drug Administration in the form of Investigational New Drug and were prepared by the Research Pharmacy of the MedStar Washington Hospital Center following specific procedures to ensure accurate bioavailability and sterility of the solutions.

### Assessment of Vascular Responses to Acetylcholine, Sodium Nitroprusside and BQ-123

Endothelial function was tested as previously described in detail [11]. Briefly, forearm blood flow was measured by strain-gauge plethysmography, at baseline and after intra-arterial infusion of increasing doses of the endothelium-dependent vasodilator acetylcholine (ACh, Sigma Chemical Co., St. Louis, Missouri. Infusion rates: 7.5, 15, and 30 μg/min), of the endothelium-independent vasodilator sodium nitroprusside (SNP, Sigma Chemical Co., St. Louis, Missouri. Infusion rates: 0.8, 1.6, and 3.2 μg/min). Drugs sequence was randomized to avoid bias related to the order of infusion. Subsequently, BQ-123 (Peninsula Laboratories) was infused at 10 nmol/min (10 nmol/mL solution), a dose that effectively counteracts the vasoconstrictor effect of ET-1 infusion in the human forearm [12]. BQ-123 was given for 60 minutes (1 mL/min infusion rate) and FBF was measured every 10 minutes.

### Statistical analysis

Two means were compared by Student’s t test. Within each group, changes in FBF from baseline in response to the infused drugs were assessed by 1-way ANOVA for repeated measures. Group comparisons of the responses to ACh, SNP, and BQ-123 were performed by 2-way ANOVA. Association analyses between CRP levels and responses to BQ-123 were performed using Pearson’s correlation coefficient. All calculated probability values are 2-tailed, and a probability value <0.05 was considered to indicate statistical significance. All group data are reported as mean ± SEM.

## Results

The clinical characteristics and lipid profile of the 114 participants (50 women and 64 men) included in this analysis are reported in Table 1. Among these patients, 40 (35%) had a diagnosis of hypertension, 40 (35%) were hypercholesterolemic, and 35 (30%) had type 2 diabetes. No significant sex-specific differences were observed in these values except for higher CRP and HDL levels and lower QUICKI insulin sensitivity index in women as compared to men. No significant changes were noted in the mean arterial pressure and heart rate after infusion of any of the study drugs, thus indicating that the drug effects were limited to the infused forearm and did not extend to the systemic circulation (data not shown). Baseline FBF was similar between study groups at all times (p > 0.05 for all comparisons).

**Table 1:** Baseline characteristics of study participants.

	***Women* (n = 50)**	***Men* (n = 64)**	***P Value***
*Age (years)*	52 ± 8	55 ± 9	0.19
*BMI (kg/m* ^*2*^ *)*	33.4 ± 7.3	31.2 ± 5.1	0.19
*Waist (cm)*	105 ± 15	110 ± 14	0.16
*Glucose (mg/dL)*	121 ± 48	115 ± 51	0.55
*Insulin (pmol/L)*	13.7 ± 7.2	13.3 ± 12.7	0.83
*QUICKI*	0.3200 ± 0.027	0.3329 ± 0.039	0.047
*TC (mg/dL)*	197 ± 58	189 ± 45	0.41
*LDL-C (mg/dL)*	118 ± 50	123 ± 41	0.52
*HDL-C (mg/dL)*	53 ± 15	44 ± 11	<0.001
*TGL (mg/dL)*	130 ± 100	127 ± 80	0.86
*CRP (mg/L)*	6.67 ± 7.34	2.95 ± 3.08	<0.001
*SBP (mmHg)*	133 ± 21	136 ± 26	0.61
*DBP (mmHg)*	70 ± 11	71 ± 14	0.60
*HR (beats/min)*	67 ± 11	63 ± 13	0.07
*Smoking (%)*	7 (15)	10 (16)	1.00

Values are reported as mean ± SD. BMI: body mass index; QUICKI: quantitative insulin sensitivity check index; TC: total cholesterol; LDL-C: low-density lipoprotein cholesterol; HDL-C: high-density lipoprotein cholesterol; TGL: triglycerides; CRP: C-reactive protein; SBP: systolic blood pressure; DBP: diastolic blood pressure; HR: heart rate.

### Vascular Responses to Acetylcholine in Women and Men

**Fig 1:**
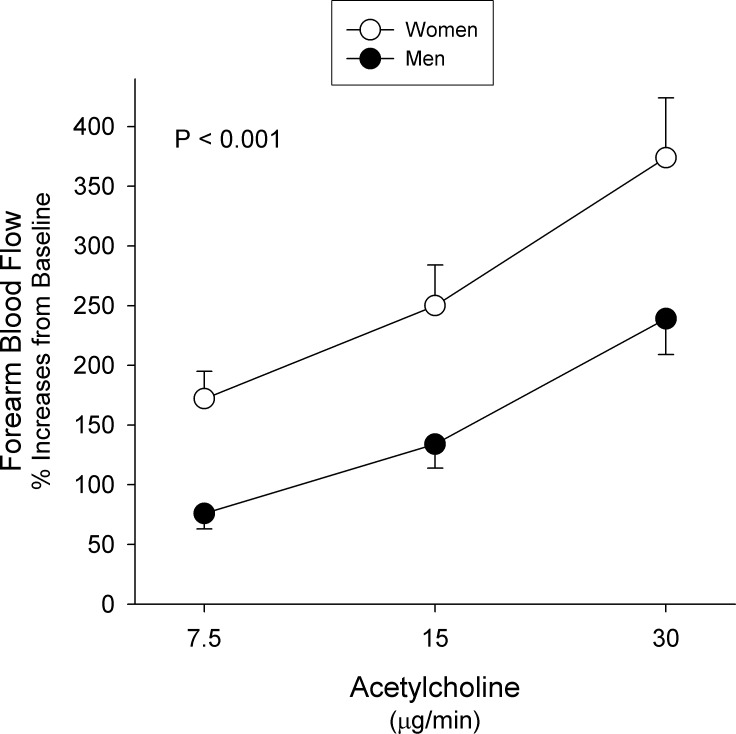
Responses to Acetylcholine. Graph showing forearm blood flow responses to increasing doses of acetylcholine in women (white dots) and men (black dots) with cardiovascular risk factors. Values represent mean±SEM. The probability value refers to the comparison between the 2 groups in forearm blood flow response to acetylcholine from baseline by 2-way ANOVA.

Acetylcholine infusion caused a progressive significant increase in FBF from baseline in both women (p < 0.001) and men (p < 0.001). When the FBF responses were compared between the two groups, women had a greater vasodilator response to ACh as compared to men (Fig 1). As shown in Table 2, subgroup analysis according to diagnosis showed that women with hypertension had a significantly greater vasodilator response to ACh as compared to men (p = 0.0019); however, in the diabetic or hypercholesterolemic patients no difference was observed between the two sex groups (p = 0.31 and p = 0.16, respectively).

**Table 2:** Percent increases in forearm blood flow from baseline in response to acetylcholine infusion in the three diagnosis subgroups.

*Subgroup*	*Sex*	*Percent Increase in Forearm Blood Flowfrom Baseline*			*P Value*
		***ACh 7*.*5***	***ACh 15***	***ACh 30***	
***Hypertension***	Women (n = 12)	259 ± 183	355 ± 273	502 ± 379	0.0019
	Men (n = 28)	62 ± 96	139 ± 168	230 ± 247	
***Hypercholesterolemia***	Women (n = 19)	168 ± 160	232 ± 202	350 ± 361	0.31
	Men (n = 21)	95 ± 101	131 ± 145	265 ± 264	
***Diabetes***	Women (n = 19)	118 ± 139	201 ± 238	313 ± 77	0.16
	Men (n = 15)	74 ± 110	131 ± 159	220 ± 52	

Values are reported as mean ± SD. ACh: acetylcholine.

### Vascular Responses to Sodium Nitroprusside in Women and Men

**Fig 2:**
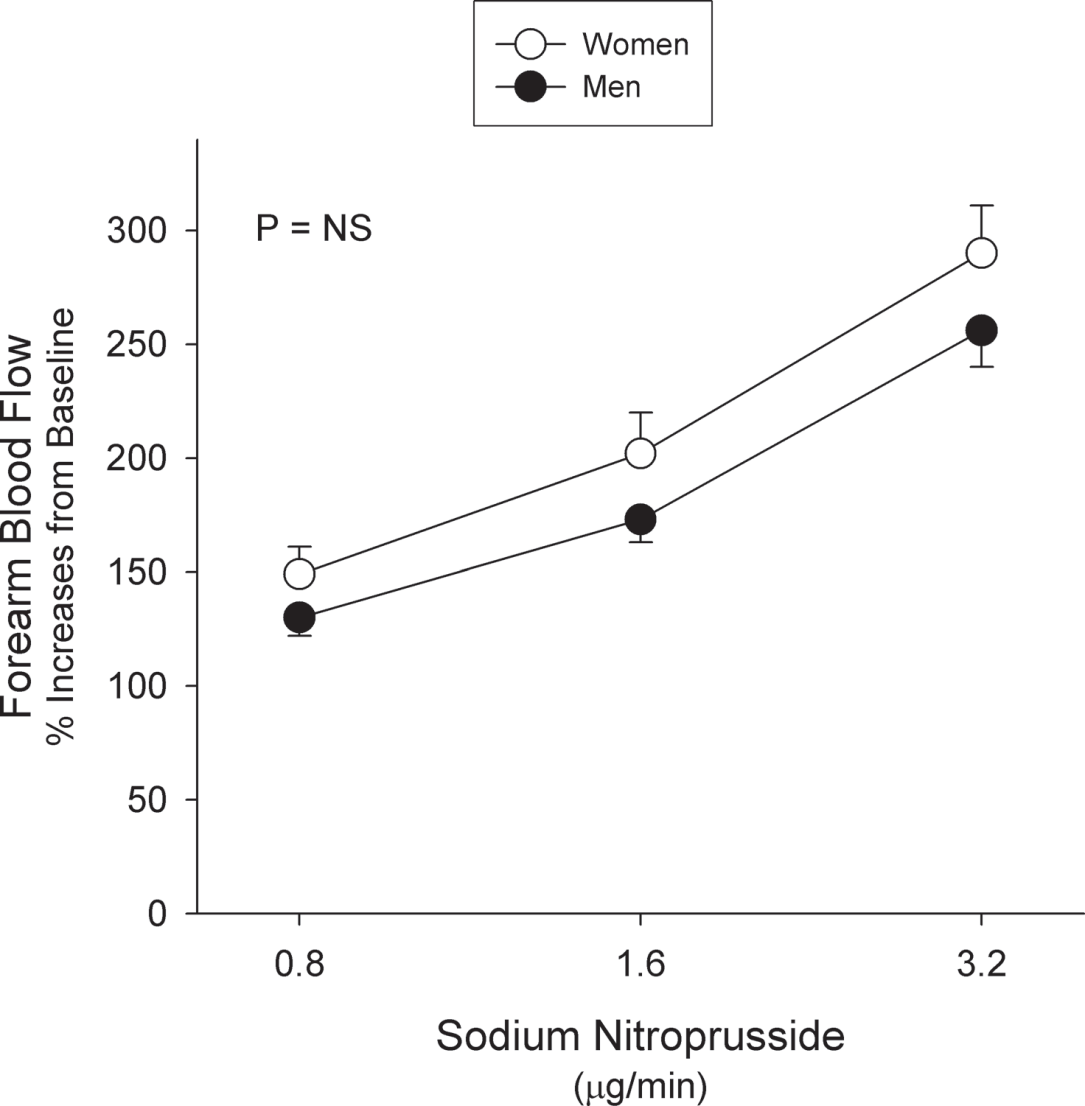
Responses to Sodium Nitroprusside. Graph showing forearm blood flow responses to increasing doses of sodium nitroprusside in women (white dots) and men (black dots) with cardiovascular risk factors. Values represent mean±SEM. The probability value refers to the comparison between the 2 groups in forearm blood flow response to sodium nitroprusside from baseline by 2-way ANOVA.

In both women and men, infusion of SNP was associated with a significant increase in FBF compared to baseline (p < 0.01 for both). When these responses were compared between the two groups, no significant differences in the vasodilator responses to SNP were observed (Fig 2). Subgroup analysis according to diagnosis showed no significant differences in SNP-induced vasodilation when compared between women and men in any of the subgroups (p > 0.05 for all).

### Vascular Responses to ET_A_ Receptor Blockade in Women and Men

**Fig 3:**
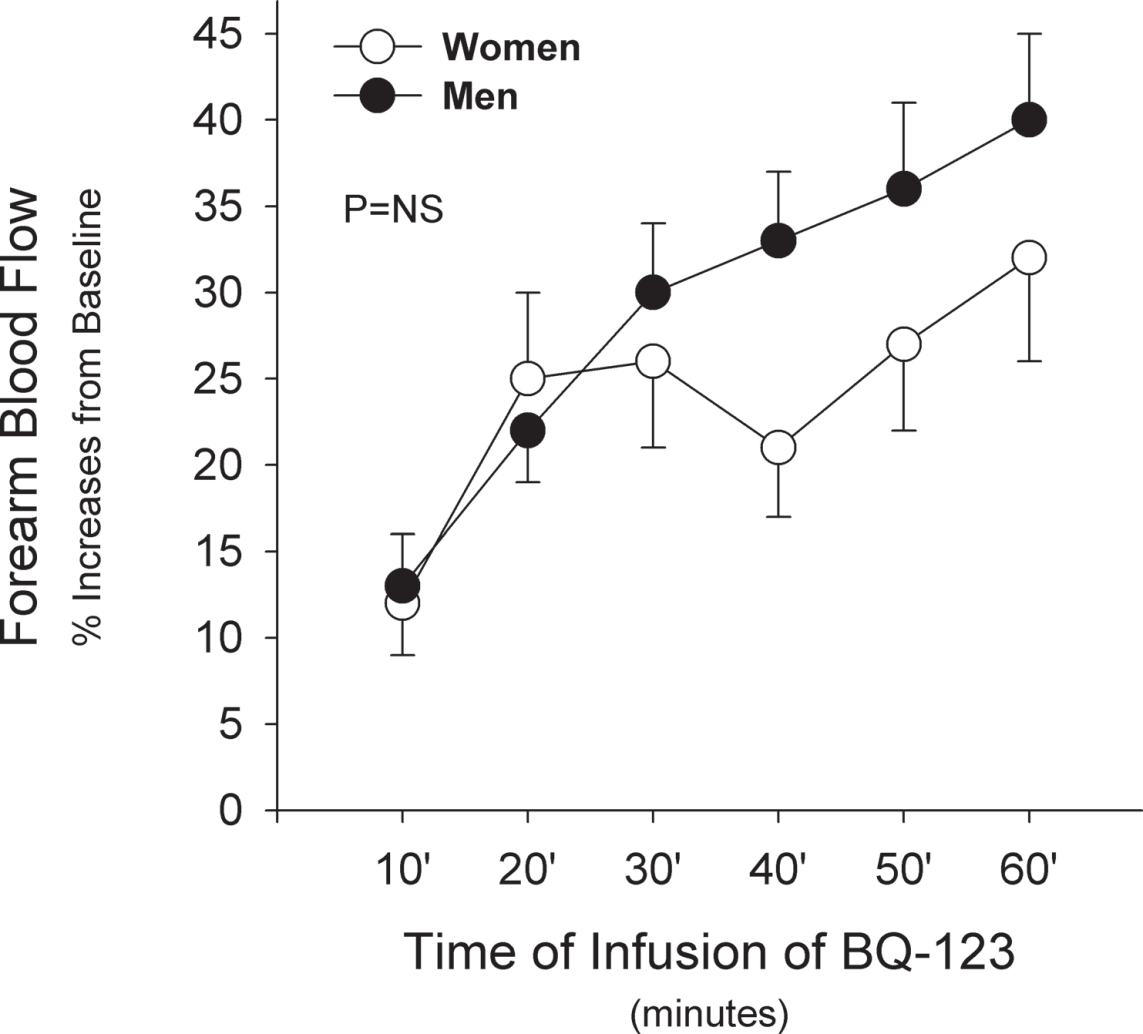
Responses to BQ-123. Graph showing forearm blood flow responses to infusion of BQ-123 (10 nmol/min) in women (white dots) and men (black dots) with cardiovascular risk factors. Values represent mean±SEM. The probability value refers to the comparison between the 2 groups in forearm blood flow response to BQ-123 from baseline by 2-way ANOVA.

In both women and men, BQ-123 infusion resulted in a significant vasodilator response from baseline (p < 0.001 for both). When these responses were compared between the two groups, no significant differences were noted (Fig 3). Subgroup analysis according to diagnosis showed similar results between women and men in the three diagnostic groups (p > 0.05 for all).

### Vascular Response to ET_A_ Receptor Blockade and CRP levels

ET-1 gene expression is induced by several factors including inflammatory mediators. As women in our study had higher levels of CRP than men, we conducted specific analyses to ascertain a potential contribution of this inflammatory marker to vascular ET-1 activity. No statistically significant correlations between CRP levels and the vasodilator responses to BQ-123 infusion at 60 minutes were observed in the whole population (r = 0.10, p = 0.23) and in any of the sex groups (r = 0.16, p = 0.27 and r = 0.13, p = 0.31 in women and men, respectively).

## Discussion

The main findings of our investigation are that, in middle-aged post-menopausal women and men with CRFs, the vasodilator response to intra-arterial infusion of ACh is significantly greater in women as compared to men; however, no significant differences exist in the response to the infusion of SNP and to selective ET_A_ receptor blockade. These findings suggest that the presence of CRFs is associated with sex-specific effects on endothelial function.

### Impact of CRFs on Endothelium Dependent Dilation

The results of our study for the first time expand the previous observations by Celermajer and colleagues [8], who showed a delayed age-related decline of NO-mediated vasodilation in healthy women compared to healthy men, such that, after age 40 years and up to the mid 60’s, women have better endothelial vasodilator function than their male counterparts. In conjunction with those observations, the present study findings would suggest that the impact of CRFs is such that the differential advantage in EDD observed in healthy women compared to that of healthy men is maintained. Of note, we found no differences in the endothelium-independent responses to SNP between men and women with CRFs. This finding helps to confirm the notion that changes in vascular reactivity are not related to potential sex-specific differences in the smooth muscle sensitivity to NO and, hence, are secondary to endothelium-specific effects of CRFs. Our findings are in agreement with those recently reported by Schnabel and colleagues [13], who observed higher flow-mediated dilation in postmenopausal women with CRFs as compared with men. However, in our investigation EDD was defined as the response to intra-arterial infusion of ACh in the forearm, suggesting that also in the microcirculation CRFs do not abrogate the sex advantage in endothelial vasodilator function.

### Impact of CRFs on Endothelin Activity

Our finding that no significant differences exist in vascular ET-1 activity between women and men with CRFs expands the recent report by Stauffer and colleagues, who showed that healthy middle-aged and older women have lower forearm ET-1 activity as compared to men [9]. On average, women and men participating in our study were slightly younger than those on Stauffer et al’s report, but appeared to mainly differ for the presence of CRFs. Taken together, the findings of both investigations indicate that, compared with men, healthy women have lower ET-1 vasoconstrictor activity, even in their mid to late 50’s. However, the presence of CRFs is associated with a loss of this benefit, suggesting a more prominent increase of ET-1 activity in women than in men.

### Sex-specific effects of CRFs on Endothelial Function

In several previous investigations, we have shown that various CRFs are associated with impaired EDD to ACh and other vasodilator agents [11, 14]. This impairment has been linked to numerous pathogenetic mechanisms such as increased oxidative stress with excessive NO degradation by free radicals, abnormal intracellular pathways with deficient activation of endothelial NO synthase (eNOS), and eNOS uncoupling with decreased NO production [15]. The evidence that post-menopausal women with CRFs maintain a higher EDD compared to men suggests that the overall effect of CRFs on endothelial vasodilator function is similar across sexes and that the same mechanisms outlined above are contributing to the impairment of EDD. The differential advantage observed in women may be related to the lasting endothelial effects of estrogens in the early postmenopausal period. In support of this hypothesis, Moreau et al. in a recent paper showed that the decline in EDD across stages of the menopause transition in healthy women is progressive [16], so that women in early post-menopause like our population may still have residual protective effects from estrogens. Importantly, these authors also showed that the effects of estrogen on endothelial function are independent of traditional CRFs, suggesting that the presence of CRFs does not abrogate the protective vascular actions of these hormones.

In addition to an abnormal vasodilator function, we have demonstrated that patients with hypertension [4], hypercholesterolemia [5], and type 2 diabetes [6] have higher vascular ET-1 activity, likely due to increased ET-1 gene expression triggered by a host of factors including angiotensin 2, insulin, oxidized LDL, and inflammatory cytokines [17]. In the present study, in contrast to the response to ACh, women and men with CRFs showed similar vasodilation to ET_A_ receptor blockade. This evidence suggests that, in healthy women, the mechanisms underlying their higher EDD differ from those accounting for the lower ET-1 activity. In particular, the ET-1 system appears to be more susceptible to the endothelial effects of CRFs than the NO pathway.

Of note, we have previously shown that ET-1 receptor blockade improves vasodilator responses to ACh in patients with hypertension[18]. That observation indicates that an increased ET-1 activity may contribute to the abnormal EDD observed in patients with CRFs. Thus, it would seem intuitive that a better endothelium-dependent reactivity to ACh is accompanied by a lower vascular ET-1 activity. However, significant methodological differences are present between our two investigations, which may underlie the apparent discrepancy noted. In our previous study, the interaction between the endothelial NO pathway triggered by ACh and the ET-1 system was tested using combined ET_A_ and ET_B_ receptor blockade with co-infusion of BQ-123 and BQ-788, respectively, whereas in our current investigation we focused on selective ET_A_ blockade with BQ-123. As in patients with hypertension dual ET_A_ and ET_B_ antagonism is associated with additional vasodilator effect compared with selective ET_A_ blockade [4] it is possible that lack of difference is a reflection of the use of selective ET_A_ versus dual ET_A_ and ET_B_ antagonism. Additionally, the present study was a retrospective analysis and was not designed to specifically investigate the interactions between the NO and the ET-1 system in women and men.

### Effects of Diagnosis on the Impact of CRFs on Endothelial Function

In our study, we included women and men with different CRFs, namely hypertension, hypercholesterolemia and type 2 diabetes, to investigate a group of patients representative of the general population. It is however to be noted that type 2 diabetes exerts a more marked effect on cardiovascular risk in women than in men [19] and may be associated with specific effects on endothelial function. Our subgroup analyses according to diagnosis show that EDD is significantly higher in women than men only in patients with hypertension. While one could expect that the higher risk in diabetic women is associated with worse endothelial function compared to men, it is not necessarily the case as the increased risk may reflect less aggressive treatment and poorer control of risk factors in diabetic women compared to men [20, 21]. This finding may indicate a possible role of diagnosis in the observed sex differences in EDD. However, the number of patients included in the analyses was small and a lack of power may account for the non-significant differences observed in participants with hypercholesterolemia and in those with diabetes. In contrast, consistent with the main analyses, no sex differences in SNP-induced vasodilation and ET-1 activity were observed in any of the diagnosis subgroups.

### Study Limitations

A number of limitations need to be acknowledged. First, our study is a retrospective analysis of prospectively collected data; thus, effects of uncontrolled and potentially confounding variables cannot be excluded. Second, our study participants comprised middle-aged women and men and the validity of our results may be limited to this age population. Third, because of the technique employed, more detailed insights into the mechanisms underlying the effects of CRFs on endothelial function cannot be derived from these observations.

### Conclusions

Middle-aged women with CRFs have significantly higher EDD than middle-aged men; however, vascular ET-1 activity is similar in the two groups. These findings suggest that the presence of CRFs is associated with sex-specific effects on endothelial function. Further study is needed to confirm our findings and to characterize the mechanisms underlying this sex-specific regulation of endothelial function.
